# The impact of the COVID-19 pandemic on the lives of the 2004 Pelotas (Brazil) birth cohort adolescents

**DOI:** 10.1590/0102-311XEN063724

**Published:** 2025-06-20

**Authors:** Alicia Matijasevich, Jessica Mayumi Maruyama, Iná S. Santos, Alana Carolina Andrade Dalla, Aluísio J. D. Barros, Luciana Tovo-Rodrigues

**Affiliations:** 1 Departamento de Medicina Preventiva, Faculdade de Medicina da Universidade de São Paulo, São Paulo, Brasil.; 2 Universidade Presbiteriana Mackenzie, São Paulo, Brasil.; 3 Programa de Pós-graduação em Epidemiologia, Universidade Federal de Pelotas, Pelotas, Brasil.

**Keywords:** COVID-19 Pandemic, Adolescent, Cohort Studies, Pandemia por COVID-19, Adolescente, Estudos de Coortes, Pandemia de COVID-19, Adolescente, Estudios de Cohortes

## Abstract

Insights into the experiences of adolescents during the COVID-19 pandemic are crucial for understanding the extent of their impact and guiding future responses to similar challenging events. This study aims to describe the effects of the pandemic on different aspects of adolescents’ lives and examine how they varied according to socioeconomic, family, and individual characteristics. We used data from a subsample of the 2004 Pelotas (Brazil) birth cohort to investigate the reported impacts of the COVID-19 pandemic on adolescents (N = 1,806; mean age = 17.41, range: 16.73-17.95). We investigated a range of issues and impacts caused by the pandemic and social distancing measures, considering their influence on various aspects of life. Additionally, we evaluated a spectrum of socioeconomic, family, and individual variables. Data were collected via in-person interviews from August to December 2021. Roughly 20% of adolescents have reported significant disruptions in their lives due to the COVID-19 pandemic. The effects of the pandemic were more pronounced among females, private school attendees, non-beneficiaries of the government cash transfer benefit (Brazil Assistance - *Auxílio Brasil*), and adolescents who reported having a regular or poor health status. Adolescents have shared a multitude of stressors and concerns directly linked to the pandemic, underscoring a notable trend of heightened impacts among female adolescents. The COVID-19 pandemic has had far-reaching consequences on various aspects of adolescents’ daily lives. Recognizing the perspectives of adolescents in this challenging period is crucial for effectively addressing their needs in the post-pandemic society.

## Resumo

Conhecimentos sobre as experiências de adolescentes durante a pandemia da COVID-19 são cruciais para entender a extensão do impacto destas experiências e orientar respostas futuras a eventos desafiadores semelhantes. Este estudo tem como objetivo descrever os efeitos da pandemia em diferentes aspectos da vida dos adolescentes e examinar como esses impactos variaram de acordo com características socioeconômicas, familiares e individuais. Usamos dados de uma subamostra da coorte de nascimentos de Pelotas, Rio Grande do Sul, Brasil, de 2004 para investigar os impactos da pandemia da COVID-19 em adolescentes (N = 1.806; média de idade = 17,41, variação: 16,73-17,95). Examinamos uma série de questões e impactos resultantes da pandemia e das medidas de distanciamento social, considerando sua influência em vários aspectos da vida. Além disso, avaliamos um espectro de variáveis socioeconômicas, familiares e individuais em nossa avaliação. Os dados foram coletados por meio de entrevistas presenciais de agosto a dezembro de 2021. Aproximadamente 20% dos adolescentes relataram interrupções significativas em suas vidas devido à pandemia. Os efeitos da pandemia foram mais acentuados entre o sexo feminino, estudantes de escolas privadas, não beneficiários do Auxílio Brasil e adolescentes que reportaram estado de saúde regular ou ruim. Os adolescentes compartilharam uma infinidade de fatores de estresse e preocupações diretamente ligados à pandemia, ressaltando uma tendência notável de impactos intensificados entre as adolescentes do sexo feminino. A pandemia da COVID-19 teve consequências de longo alcance em vários aspectos da vida cotidiana dos adolescentes. Reconhecer as perspectivas dos adolescentes neste período desafiador é crucial para atender efetivamente às suas necessidades na sociedade pós-pandemia.

## Resumen

El conocimiento de las experiencias de los adolescentes durante la pandemia de la COVID-19 es fundamental para comprender el alcance del impacto de estas experiencias y para orientar las respuestas futuras a eventos desafiantes similares. Este estudio tiene como objetivo describir los efectos de la pandemia en diferentes aspectos de la vida de los adolescentes y examinar cómo variaron estos impactos según las características socioeconómicas, familiares e individuales. Utilizamos datos de una submuestra de la cohorte de nacimiento en Pelotas, Rio Grande do Sul, Brasil, 2004 para investigar los impactos de la pandemia de la COVID-19 en adolescentes (N = 1.806; edad media = 17,41, rango: 16,73-17,95). Analizamos una serie de problemas e impactos resultantes de la pandemia y de las medidas de distanciamiento social desde su influencia en diversos aspectos de la vida. Además de evaluar las variables socioeconómicas, familiares e individuales. Para analizar los datos se realizaron entrevistas presenciales de agosto a diciembre de 2021. Aproximadamente el 20% de los adolescentes informaron haber sufrido significativas cambios en sus vidas debido a la pandemia. Los efectos de la pandemia fueron más frecuentes entre las mujeres, los estudiantes de escuelas privadas, los no beneficiarios del programa gubernamental Auxilio Brasil y los adolescentes que tenían un estado de salud regular o deficiente. Los adolescentes reportaron diversos factores estresantes y preocupaciones directamente relacionadas con la pandemia, lo que subraya una tendencia notable de impactos más intensificados entre las adolescentes. La pandemia de la COVID-19 ha tenido consecuencias a largo plazo en diversos aspectos de la vida cotidiana de los adolescentes. Reconocer las perspectivas de los adolescentes en este período desafiante es crucial para satisfacer eficazmente sus necesidades en la sociedad pospandemia.

## Introduction

The outbreak of COVID-19 was declared a pandemic by the World Health Organization (WHO) on March 11th, 2020 ^1^. After that, several public health measures were imposed by most countries to contain the spread of the virus, abruptly changing the population’s daily routine, including work, social, and personal life. Despite governmental efforts to reduce the contagion and mortality rates, there have been millions of deaths globally ^2^. In Brazil, several political and economic factors contributed to lead the country to the highest absolute number of COVID-19-deaths in October of 2021 ^3^. Now, the pandemic has been on a downward trend with the increased immunity of the population from vaccination and previous infection, resulting in fewer deaths. This scenario led the WHO to declare the COVID-19 over as a global health emergency on March 5th, 2023 ^4^. However, many of the potential prolonged consequences of the intense stress caused in the population by three years of pandemic are still under investigation by the scientific community.

The disruptions caused by the COVID-19 pandemic were expected to have a great impact on individuals’ well-being and mental health, but, for adolescents and young adults, the pandemic may have also affected their development in several domains with potential long-term consequences. School closures and restrictions on in-person interactions with peers have a great influence on adolescent’s social development and identity formation ^5^. The cancellation or remote classes vastly adopted by many institutions may repercuss in the learning process expected for this stage of life, with potential impairments in school achievements and future job prospects ^6^. The stay-at-home orders and lockdowns forced adolescents to spend more time with the family, which despite creating an opportunity to improve communication and redefining family ties, also had the potential to increase the chances of more conflicts with parents ^70^. The uncertainties of living during a pandemic and the unpredictability of the future can lead to opportunities of self-awareness and coping strategies but also trigger symptoms of anxiety ^8^. The economic recession faced by all countries, with job losses and financial difficulties, is also an influential stressor with direct and indirect effects for adolescents’ well-being ^9^. In fact, the occurrence and interaction of such factors may be associated with the longitudinal deterioration of adolescents’ mental health before and during the pandemic, as reported by recent systematic reviews and meta-analysis ^7^ ^,^ ^10^. Nonetheless, emerging literature has shown the impact of the pandemic may be heterogeneous across population subgroups, and studies indicate that some adolescents reported an improvement in mental health indicators and positive development during this period ^11^ ^,^ ^12^. 

Beyond the quantification of the COVID-19 pandemic effects on well-being, it is also important to understand the adolescents’ perception of the challenges and worries during this period. Qualitative research, along with quantitative studies, showed the adolescents’ experiences varied widely ^13^ ^,^ ^14^ ^,^ ^15^ ^,^ ^16^. The negative impacts of the pandemic described by the adolescents over their lives include concerns related to remote schooling, loneliness, unpredictability of future events, and fear of losing important moments of their lives ^13^ ^,^ ^14^ ^,^ ^15^ ^,^ ^16^. On the other hand, they also described positive changes associated to the pandemic, as decreased stress from more flexible schedules, strengthened family bonds, and more opportunities to self-development and self-discovery ^11^ ^,^ ^14^ ^,^ ^16^. In addition to the expected differences from individual perspectives, divergences on the experiences of the adolescents during the pandemic may also be influenced by geographical, cultural, and socioeconomic aspects ^16^ ^,^ ^17^. A study ^17^ including adolescents from Luxembourg, Germany, and Brazil showed that fear of illness was the strongest predictor of lower emotional well-being in the three countries. However, older adolescents experienced more negative emotions only in Brazil ^17^. Furthermore, the authors ^17^ also reported that participants from Luxembourg and Germany were more similar to each other on some demographic and pandemic indicators than participants from Brazil. For example, Brazilian adolescents reported more frequently not having access to a personal or tablet computer, not having an own bedroom, and being single child when compared to their European peers ^17^. A study from Shuka et al. ^18^ also reported cross-cultural differences in psychological well-being during the pandemic when comparing adolescents from India, Israel, and the United Kingdom. The authors ^18^ discuss that such differences may be associated with the underlying sociocultural values disparities within the three countries, such as individualistic versus collectivist society values. Although there are distinct findings across and within countries, most studies with adolescents were conducted in the Global North ^7^ ^,^ ^10^, and more representative and diverse samples are essential to fully understand the youth’s needs in terms of global health. 

Considering the effects of the pandemic on youth’s well-being can be greatly divergent, it is fundamental to consider adolescents’ experiences by their own perception. Although children and adolescents have the right to be heard, as stated by the United Nations Convention on the Rights of Child (1989) ^19^, several decisions related to youth’s lives are made without their opinion on the matter ^10^ ^,^ ^18^. For this purpose, descriptive epidemiological studies are essential and useful tools to advance in the goals to ensure equity in health ^20^ ^,^ ^21^. As Fox et al. ^22^ reported, the COVID-19 outbreak resumed and highlighted the role of descriptive studies in providing evidence in a public health crisis context and informing subsequent research. 

Despite the robust body of research on the impact of the COVID-19 pandemic on adolescents’ well-being, most studies have focused on high-income countries and there are limited data from low- and middle-income countries such as Brazil. Additionally, while many investigations have examined mental health outcomes and educational disruptions, fewer studies have considered adolescents’ own perceptions of how the pandemic affected various aspects of their lives. To address such gap, this study aimed to describe the self-reported impacts of the COVID-19 pandemic on multiple life domains among adolescents from a Brazilian birth cohort. Furthermore, we explored how the perceived effects varied according to socioeconomic, family, and individual characteristics. By centering the perspectives of the adolescents, our study provides a more nuanced understanding of their challenges and needs, offering crucial insights to inform future policies and interventions aimed at mitigating long-term adverse outcomes in this age group.

## Methods

### Participants and procedure

The study sample comprises the adolescents belonging to the 2004 Pelotas birth cohort, a prospective and population-based birth cohort from Brazil. Mothers of all children born in 2004 were invited to participate in the cohort, which included 4,231 participants, with a non-response rate at recruitment lower than 1%. In addition to the perinatal interview, there were follow-up waves at mean ages (standard deviation - SD) 3.0 (0.1), 11.9 (0.2), 23.9 (0.4), and 49.5 (1.7) months (at the participants’ home), and at 6.8 (0.3) and 11.0 (0.3) years (at a research clinic). The seventh follow-up, at a mean age of 15.7 years (0.2), occurred from November 2019 to March 2020, when the data collection at the research clinic had to be interrupted due to the COVID-19 pandemic. At that point 1,949 adolescents and their caregivers were interviewed, corresponding to 47.8% of the original cohort. The peri-pandemic wave, which occurred from August to December, 2021, aimed to interview this same subsample, enabling the assessment of the mental health impacts of COVID-19 pandemic by providing data from immediately before and during the pandemic. In addition, the peri-pandemic wave also assessed several pandemic-specific factors that may be associated with health and well-being outcomes in the cohort participants. Further information about the cohort and procedures were published elsewhere ^23^ ^,^ ^24^.

### Ethics

All the 2004 Pelotas (Brazil) birth cohort follow-ups were approved by the Federal University of Pelotas, Medical School Research Ethics Committee (35/10,889.753/CAAE: 38013414.9.0000.5317 and 3.554.667/CAAE: 20183419.1.0000.5317). All main caregivers and adolescents signed an informed consent form before data collection. This study was also approved by the Ethics Committee for Analysis of Research Projects (CAPPesq; Research protocol n. 4.951.457) of the Clinical Hospital of the School of Medicine of the São Paulo University (FMUSP). 

### Measures and statistical methods

The questionnaire used in the peri-pandemic assessment included items regarding the impacts of the COVID-19 pandemic over several aspects the adolescents’ lives. The first item asked, “How much do you believe the pandemic/social distancing measures affected your life?” and the possible answers were “I was not affected”, “I was a bit affected”, “I was moderately affected”, and “I was greatly affected”. For those who did not answer “not affected”, 19 follow-up close-ended questions were asked. Such questions addressed possible ways the pandemic/social distancing measures affected the adolescents’ lives, including fear of illness, screen time, financial problems, eating, and sleep patterns. 

Other pandemic-specific questions were also asked in the peri-pandemic wave, such as schooling during lockdown, maternal job loss, COVID-19 infection, weight changes, level of social distancing restrictions adopted in 2020 and 2021 (“no or low restrictions”, “moderate restrictions”, and “strict restrictions”), and whether the family received the government transfer benefit during the pandemic (Brazil Assistance). In addition, we asked two questions related to schooling and classes during the pandemic. One of the questions was: “During the first year of the pandemic (2020), did you have in-person or online classes?” and the possible answers were “I was not enrolled in school”; “No, the classes were cancelled”; “Yes, only in-person classes”; “Yes, only virtual classes”, and “Yes, both in-person and virtual classes”. The same question was asked concerning the second year of the pandemic (2021). The adolescents’ sex and maternal skin color were assessed at the perinatal interview. Adolescents’ religiosity was assessed in the pre-pandemic wave with the question “Do you have any religion?”, with yes/no answers. 

Descriptive and bivariate analyses (frequencies, χ^2^ tests) were used to examine self-reported impact of the pandemic across groups, which were defined by socioeconomic, family, and individual characteristics. To examine the factors associated with reporting a great impact of the pandemic, we performed a binary logistic regression analysis. The dependent variable was categorized as 1 = “greatly affected” and 0 = “not affected/little affected/ moderately affected”. Independent variables included the abovementioned sociodemographic factors and pandemic-related conditions. Age was included as a covariate in the analysis. Odds ratios (OR) and 95% confidence intervals (95%CI) were estimated to assess the strength of association between each predictor and the likelihood of reporting a great impact of the pandemic. This was a complete case analysis. Missing observations for each variable were excluded from their respective analyses. The percentage of missing observations varied from 0.00 to 1.02%. The data were analyzed using Stata version 14.20 (https://www.stata.com).

## Results

### Sample description

A total of 1,805 adolescents (mean age = 17.4, range: 16.73-17.95, 95%CI: 17.4-17.4) and their caregivers were interviewed in person during the peri-pandemic assessment, which corresponded to 92.6% of the target subsample from the pre-pandemic wave. Male adolescents corresponded to 50.8% of the sample. Most adolescents enrolled in a school studied at public institutions (89.4%). A total of 226 (14.7%) adolescents from public schools and 10 (5.5%) from private schools affirmed that classes were cancelled in 2020 due to the pandemic. In 2021, these numbers declined to 2.9% (n = 45) among adolescents from public schools and 1.1% (n = 2) among adolescents from private schools. The number of adolescents that were reported to be outside the school system rose from 5% in 2020 to 11% in 2021. By the time of data collection (August to December 2021), 14.5% of the adolescents reported a previous COVID-19 infection. Table 1 describes further sample characteristics.

**Table 1:** Descriptive analysis of the included sample (N = 1,806).

Characteristics	n (%) or mean (95%CI)
Age [years] (range: 16.73-17.95)	17.41 (17.40-17.43)
Sex	
Male	925 (50.85)
Female	894 (49.15)
Type of school	
Public	1,529 (89.42)
Private	181 (10.58)
Not enrolled in school	
First year of the pandemic (2020)	91 (5.05)
Second year of the pandemic (2021)	199 (11.04)
No classes during the pandemic	
First year of the pandemic (2020)	237 (13.04)
Second year of the pandemic (2021)	52 (2.88)
Beneficiary of Brazil Assistance	
Yes	860 (47.65)
No	945 (52.35)
Family’s financial situation compared to the pre-pandemic period	
Worst	997 (55.24)
Equal	124 (6.87)
Better	684 (37.89)
Maternal job loss during the pandemic	
Yes	367 (20.32)
No	1,439 (79.68)
Maternal skin color	
White	1,339 (73.33)
Black/Mixed-race	487 (26.67)
Number of people living in the house *	
2-3	608 (35.66)
4-5	896 (52.55)
≥ 6	201 (11.79)
COVID-19 infection **	
Yes	262 (14.53)
No	1,541 (84.47)
Religion	
Yes	852 (46.66)
No	974 (53.34)
Social distancing restrictions in 2020 and 2021	
No or low social distancing	215 (11.94)
Moderate social distancing	577 (32.04)
Strict social distancing	1,009 (56.02)
Perceived health status	
Excellent	344 (19.09)
Very good/Good	1,151 (63.87)
Regular/Poor	307 (17.04)
Weight change during lockdown	
Lost weight	378 (20.97)
Gained weight	896 (49.69)
Stayed at the same weight	432 (23.96)
Did not know	97 (5.38)

95%CI: 95% confidence inteval.

* Number of people living in the house including the adolescent;

** Self-reported question at the time of assessment (August to December 2021).

Adolescents’ perception of the COVID-19 pandemic impacts over their lives according to socioeconomic, family, and individual factors

Table 2 shows that several participants’ characteristics were associated with being more affected by the COVID-19 pandemic and the social distancing measures. Overall, 355 (19.7%, 95%CI: 17.9-21.6) adolescents reported that they were greatly affected by the pandemic. A higher proportion of female adolescents reported being greatly affected by the pandemic than male adolescents. Regarding schooling variables, a higher proportion of adolescents from private schools, those who had either remote or in-person classes in 2020 and in 2021 affirmed they were more affected by the pandemic. Regarding socioeconomic factors, a more frequent report of great pandemic impact was found in adolescents from families that did not receive the federal benefit (Brazil Assistance) and from white mothers. Adolescents living in a house with two to three people reported more impacts related to the COVID-19 pandemic. Concerning individual characteristics, a stricter social distancing adopted by the adolescent was associated with a more prevalent report of being greatly impacted. Additionally, a more prevalent report of great pandemic impact was found in adolescents who reported having a regular/poor health status, and adolescents who reported gaining weight during the pandemic. Adolescents’ perception of the impacts of the pandemic over their lives did not vary according to family’s financial situation compared to the pre-pandemic period, maternal job loss during the pandemic, getting infected with COVID-19, or adolescent religiosity (Table 2). 

**Table 2:** Adolescents’ perception of the COVID-19 pandemic impacts over their life, stratified by socioeconomic, family, and individual characteristics.

Characteristcs	Not affected		Little affected		Moderately affected		Greatly affected		p-value
	n	% (95%CI)	n	% (95%CI)	n	% (95%CI)	n	% (95%CI)	
Total sample	267	14.8 (13.3-16.5)	630	35.0 (32.8-27.2)	548	30.4 (28.3-32.6)	355	19.7 (17.9-21.6)	
Sex									< 0.001
Male	157	17.2 (14.9-19.8)	340	37.3 (34.2-40.5)	268	29.4 (26.5-32.4)	147	16.1 (13.9-18.6)	
Female	110	12.4 (10.4-14.7)	290	32.7 (29.6-35.8)	280	31.5 (28.5-34.7)	208	23.4 (20.7-26.3)	
Type of school									< 0.001
Public	231	15.1 (13.4-17.0)	548	35.9 (33.5-28.3)	458	30.0 (27.7-32.3)	290	19.0 (17.1-21.0)	
Private	8	4.4 (2.2-8.6)	47	26.0 (20.1-32.9)	75	41.4 (34.4-48.8)	51	28.2 (22.1-35.2)	
Classes during the pandemic during the first year of the pandemic (2020)									< 0.001
Not enrolled in school	28	30.8 (22.1-41.0)	34	37.4 (28.0-47.8)	15	16.5 (10.1-25.6)	14	15.4 (9.3-24.4)	
No	30	12.7 (9.0-17.6)	89	37.7 (31.7-44.1)	75	31.8 (26.1-38.0)	42	17.8 (13.4-23.2)	
Yes (both remote and in-person)	209	14.2 (12.5-16.1)	507	34.4 (32.0-36.9)	458	31.1 (28.8-33.5)	299	20.3 (18.3-22.4)	
Classes during the second year of the pandemic (2021)									< 0.001
Not enrolled in school	52	26.1 (20.5-32.7)	67	33.7 (27.4-40.5)	47	23.6 (18.2-30.0)	33	16.6 (12.0-22.4)	
No	12	23.1 (13.5-36.5)	27	51.9 (38.4-65.2)	9	17.3 (9.2-30.2)	4	7.7 (2.9-18.9)	
Yes (both remote and in-person)	203	13.1 (11.5-14.9)	536	34.6 (32.3-37.0)	492	31.8 (39.5-34.1)	318	20.5 (18.6-22.6)	
Beneficiary of Brazil Assistance									< 0.001
Yes	150	17.8 (15.4-20.6)	317	37.7 (34.5-41.0)	239	28.4 (25.5-31.6)	135	16.0 (13.7-18.7)	
No	112	11.9 (10.0-14.2)	308	32.8 (29.9-35.9)	301	32.1 (29.2-35.1)	217	23.1 (20.5-25.9)	
Family’s financial situation compared to the pre-pandemic period									0.181
Worst	135	13.8 (11.7-16.1)	357	36.4 (33.5-39.5)	279	28.5 (25.7-31.4)	209	21.3 (18.9-24.0)	
Equal	107	15.8 (10.6-23.7)	224	33.2 (29.7-36.8)	226	33.5 (30.0-37.1)	118	17.5 (14.8-20.5)	
Better	20	16.1 (10.6-23.7)	43	34.7 (26.8-43.5)	37	29.8 (22.4-38.5)	24	19.3 (13.3-27.3)	
Maternal job loss during the pandemic									0.427
Yes	57	15.7 (12.3-19.9)	137	37.8 (33.0-43.0)	104	28.7 (24.3-33.6)	64	17.7 (14.7-22.0)	
No	204	14.4 (12.6-16.3)	488	34.4 (32.0-36.9)	438	30.9 (28.5-33.3)	288	20.3 (18.3-22.5)	
Maternal skin color									0.007
White	177	13.4 (11.7-15.4)	453	34.4 (31.8-37.0)	411	31.2 (28.7-33.7)	277	21.0 (18.9-23.3)	
Black/Mixed-race	90	18.7 (15.4-22.4)	177	36.7 (32.5-41.1)	137	28.4 (24.6-32.6)	78	16.2 (13.1-19.7)	
Number of people living in the house *									< 0.001
2-3	77	12.8 (10.4-15.8)	193	32.2 (28.5-36.0)	175	29.2 (25.7-32.9)	155	25.8 (22.5-29.5)	
4-5	126	14.2 (12.1-16.7)	316	35.7 (32.6-38.9)	288	32.5 (29.5-35.7)	155	17.5 (15.1-20.2)	
≥ 6	44	22.3 (17.0-28.7)	76	22.3 (17.04-28.7)	49	24.9 (19.3-31.4)	28	14.2 (10.0-19.3)	
Covid infection **									0.532
Yes	32	12.3 (8.8-16.8)	89	34.1 (28.6-40.7)	84	32.2 (26.8-38.1)	56	21.5 (16.9-26.9)	
No	235	15.3 (13.5-17.1)	541	35.1 (32.8-37.6)	464	30.1 (27.9-32.5)	299	19.4 (17.5-21.5)	
Religion									0.458
Yes	137	14.3 (12.2-16.7)	326	34.1 (31.1-37.1)	293	30.6 (27.8-33.6)	201	21.0 (18.5-23.7)	
No	130	15.4 (13.1-18.0)	304	36.1 (32.9-39.4)	255	30.2 (27.2-33.4)	154	18.3 (15.8-21.0)	
Level of social distancing restrictions									< 0.001
No or low restrictions	61	28.4 (22.7-34.8)	77	35.8 (29.7-42.5)	57	26.5 (21.0-32.8)	20	9.3 (6.1-14.0)	
Moderate restrictions	74	12.8 (10.3-15.8)	214	37.1 (33.2-41.1)	184	31.9 (28.2-35.8)	105	18.2 (15.2-21.6)	
Strict restrictions	132	13.1 (11.1-15.3)	339	33.6 (30.8-36.6)	307	30.5 (27.7-33.4)	230	22.8 (20.3-25.5)	
Perceived health status									< 0.001
Excellent	67	19.5 (15.6-21.0)	128	37.2 (32.2-42.4)	95	27.6 (23.1-32.6)	54	15.7 (12.2-19.9)	
Very good/Good	171	14.9 (12.9-17.0)	398	34.7 (31.9-37.5)	366	31.9 (29.2-34.6)	213	18.5 (16.4-20.9)	
Regular/Poor	29	9.4 (3.6-13.3)	103	33.5 (28.5-39.0)	87	28.3 (23.6-33.6)	88	28.7 (23.9-34.0)	
Weight change during lockdown									0.007
Lost weight	48	12.7 (9.7-16.5)	126	33.4 (28.8-38.3)	123	32.6 (28.1-37.5)	80	21.1 (17.4-25.6)	
Gained weight	120	13.4 (11.3-15.8)	312	34.9 (31.8-38.1)	268	30.0 (27.0-33.1)	194	21.7 (19.1-24.5)	
Stayed at same weight	73	16.9 (13.6-20.7)	158	36.6 (32.1-41.2)	132	30.56 (26.4-35.1)	69	16.0 (12.8-19.7)	
Did not know	26	26.8 (18.9-36.5)	34	35.0 (26.2-45.0)	25	25.8 (18.0-35.4)	12	12.4 (7.1-20.6)	

95%CI: 95% confidence imterval.

* Number of people living in the house including the adolescent;

** Self-reported question at the time of assessment (August to December 2021).

Table 3 shows a multivariable logistic regression analysis, in which several factors remained significantly associated with the report of being greatly affected by the pandemic. Female adolescents were more likely to report a greater impact (95%CI: 1.19-2.02, p < 0.001), compared to males. Adolescents attending private schools were also more likely to report being significantly affected (OR = 1.52, 95%CI: 1.03-2.23, p = 0.001), compared to those who attended public schools. Adolescents who reported a worse financial situation compared to the pre-pandemic period were at higher odds of being significantly affected (OR = 1.37, 95%CI: 1.03-1.81, p = 0.019). Moreover, stricter social distancing restrictions in 2020 and 2021 were associated with an increased likelihood of reporting significant impact, with ORs of 2.04 (95%CI: 1.13-3.69, p = 0.001) and 2.61 (95%CI: 1.47-4.61, p < 0.001) for moderate and strict restrictions, respectively, compared to no or low restrictions. Adolescents who were beneficiaries of the Brazil Assistance program were less likely to report a significant impact (OR = 0.65, 95%CI: 0.50-0.87, p = 0.003) compared to non-beneficiaries. Adolescents who reported a larger number of people living in their home were more likely to report being greatly affected, in which those living with four to five people had lower odds of being significantly affected (OR = 0.58, 95%CI: 0.43-0.74, p < 0.001), and those living with six or more people having the lowest odds (OR = 0.47, 95%CI: 0.29-0.78, p < 0.001), compared to those living with two to three people. Finally, adolescents who perceived their health status as regular or poor were more likely to report a greater impact (OR = 1.93, 95%CI: 1.41-2.65, p < 0.001) compared to those who perceived their health as excellent, very good, or good. The results of the multivariable analysis highlighted the effect of certain factors became marginally significant (such as maternal skin color or weight change during lockdown) or did not remain significant (such as not having classes during 2020 and 2021) after adjusting for other variables (Table 3).

**Table 3:** Multivariable logistic regression of the factors associated with reporting greatly pandemic impact on adolescents’ lives.

Characteristics	OR (95%CI)	p-value
Sex		0.001
Male	1.00 (Reference)	
Female	1.55 (1.19-2.02)	
Type of school		0.042
Public	1.00 (Reference)	
Private	1.52 (1.03-2.23)	
Classes during the first year of the pandemic (2020)		0.785
Yes (both remote and in-person)	1.00 (Reference)	
No	1.18 (0.61-2.29)	
Not enrolled in school	1.39 (0.78-2.50)	
Classes during the second year of the pandemic (2021)		0.319
Yes (both remote and in-person)	1.00 (Reference)	
No	0.88 (0.52-1.46)	
Not enrolled in school	0.31 (0.09-1.04)	
Beneficiary of Brazil Assistance		0.003
No	1.00 (Reference)	
Yes	0.65 (0.50-0.87)	
Family’s financial situation compared to the pre-pandemic period		0.019
Equal	1.00 (Reference)	
Better	1.11 (0.65-1.89)	
Worst	1.37 (1.03-1.81)	
Maternal job loss during the pandemic		0.691
No	1.00 (Reference)	
Yes	0.93 (0.67-1.31)	
Maternal skin color		0.067
White	1.00 (Reference)	
Black/Mixed-race	0.75 (0.54-1.02)	
Number of people living in the house *		< 0.001
2-3	1.00 (Reference)	
4-5	0.58 (0.43-0.74)	
≥ 6	0.47 (0.29-0.78)	
COVID-19 infection **		0.811
No	1.00 (Reference)	
Yes	1.04 (0.73-1.49)	
Religion		0.079
Yes	1.00 (Reference)	
No	1.26 (0.97-1.63)	
Social distancing restrictions in 2020 and 2021		0.001
No or low social distancing	1.00 (Reference)	
style="text-indent:12px"Moderate social distancing	2.04 (1.13-3.69)	
Strict social distancing	2.61 (1.47-4.61)	
Perceived health status		< 0.001
Excellent/Very good/Good	1.00 (Reference)	
Regular/Poor	1.93 (1.41-2.65)	
Weight change during lockdown		0.083
Stayed at the same weight/Did not know	1.00 (Reference)	
Lost weight	1.30 (0.90-1.90)	
Gained weight	1.32 (0.97-1.81)	

95%CI: 95% confidence interval; OR: odds ratio.

Note: adolescent age was included as covariate.

* Number of people living in the house including the adolescent;

** Self-reported question at the time of assessment (August to December 2021).

### Pandemic-related stressors and concerns

When asked about the concerns and worries regarding the COVID-19 pandemic, adolescents reported fear of the disease, feeling of uncertainties, and differences in sleep and eating habits. Considering the whole sample, the most frequent answers were “fear of a family member getting sick” (95.5%, 95%CI: 93.2-97.8), “My studies and learning were impaired by the school closure” (90.5%, 95%CI: 88.2-92.8), “I missed my relatives and friends” (88.3%, 95%CI: 86.0-90.6), and “I felt anxious for not knowing when the pandemic would be over” (77.3%, 95%CI: 75.0-79.6). The perception of excessive screen time was extensively reported by adolescents (74.9%, 95%CI: 72.6-77.2). A large proportion of adolescents also reported the feeling that parents were stressed (70.1%, 95%CI: 67.8-72.4), missing outdoor activities (69.6%, 95%CI: 67.3-71.9), and fear of becoming ill (68.8%, 95%CI: 66.5-71.1). Regarding the changes in eating patterns, 59.5% (95%CI: 57.2-61.8) of the adolescents reported they felt hungrier or ate more during the pandemic, whereas 21.9% (95%CI: 19.6-24,3) reported they felt less hungry or ate less. For sleep pattern, 51.5% (95%CI: 49.2-53.8) reported they felt sleepier during the pandemic, and 42.1% (95%CI: 39.8-44.4) reported having insomnia during the pandemic. Challenges concerning family relationships were also reported by the adolescents. For instance, 18.8% (95%CI: 16.5-21.1) of the adolescents reported they did not like to stay at home as they were told to help with household chores. In addition, there was no privacy at home (17.1%, 95%CI: 14.8-19.4), constant fights and conflicts (14.4%, 95%CI: 12.1-16.7), and caregiver-adolescent arguments were reported (11%, 95%CI: 8.7-13.3). Finally, 6.4% (95.5%, 95%CI: 4.1-8.7) of the adolescents felt vulnerable for not having access to masks and hygiene products (data not shown in tables).

**Figure 1:**
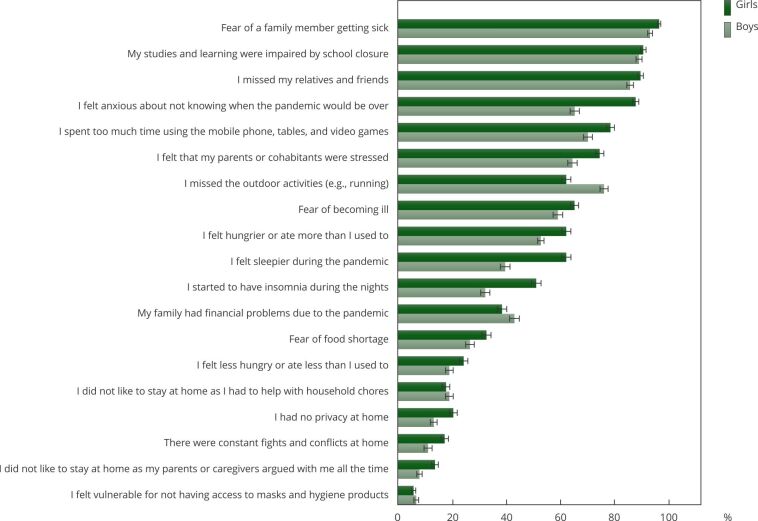
Concerns and worries related to the COVID-19 pandemic and social distancing measures reported by adolescents and stratified by sex.

Figure 1 shows the COVID-19 concerns stratified by the adolescents’ sex, with 95%CI for the proportions. Notably, a significantly higher percentage of girls reported 14 out of 19 listed items compared to boys, including “I felt anxious for not knowing when the pandemic would be over”, “I felt that my parents or cohabitants were stressed”, “I felt hungrier or ate more than I used to”, “I felt sleepier”, and “I started to have insomnia during the nights”. Missing outdoor activities and family financial problems due to the pandemic were significantly more reported by boys. There were no differences in the proportions of boys and girls in the following items: “My studies and learning were impaired by the school closure”, “I did not like to stay at home as I had to help with the household chores”, and “I felt vulnerable for not having access to masks and hygiene products” (Figure 1).

## Discussion

The current descriptive study provides insights into the perceived impacts and worries of COVID-19 pandemic over several aspects of life based on the adolescents’ report. We found that nearly one in five adolescents reported that the COVID-19 pandemic and the imposed social distancing measures greatly affected their lives. Nonetheless, almost 15% of the adolescents affirmed they were not affected by the COVID-19 pandemic. These results are in line with the studies that show an overall worsening in adolescents’ well-being during the pandemic, although this deterioration is heterogeneous and context-dependent ^11^ ^,^ ^12^ ^,^ ^25^. In fact, our findings show that the report of greater impact varies according to the socioeconomic, family, and adolescents’ characteristics.

We found that girls were 55% more likely to report being greatly affected by the COVID‐19 pandemic and social distancing when compared to boys. Moreover, a significantly higher percentage of girls reported 14 out of 19 COVID-19-related concerns compared to boys. Anxiety about the unpredictability of the pandemic, changes in sleep and eating patterns, and conflicts at home were among the stressors that affected more girls than boys. The results are in accordance with previous studies showing that girls were more severely impacted by the pandemic, especially in terms of psychological well-being ^25^ ^,^ ^26^. In the beginning of the pandemic, several researchers warned that the COVID-19 pandemic would exacerbate and highlight the existing gender inequalities in several domains of life, disproportionally affecting girls and women worldwide ^27^ ^,^ ^28^. As discussed by Mendolia et al. ^29^, the consequences of pandemic-related restrictions on gender inequality involved several domains including significant burden related to caregiving and household responsibilities, domestic violence, disrupted access to healthcare and reproductive rights, digital divide, and unemployment and loss of income. Our findings underscore the potential long-term effects of gender inequalities expanded by the pandemic, particularly on girls’ mental health. Although the immediate crisis has passed, it is essential to continue gathering knowledge on the impacts, both to address persistent inequalities and support girls’ well-being and to inform strategies that can better prepare societies for future adversities.

Regarding the school variables, students from private schools reported a great impact of the pandemic over their lives when compared to those from public schools. Importantly, the percentage of adolescents who were not enrolled in school more than doubled from the first to the second year of the pandemic. Considering the mean age of our sample, it is possible that part of this increase in the number of adolescents who were not enrolled in school is due to the high school graduation that usually occurs in this age group. However, a report from United Nations Children’s Fund (UNICEF) showed that almost two million of 11 to 19-year-old adolescents from Brazil had to drop out school during the pandemic and half of those who were not enrolled in the school had to give up studying to work and help with their families’ finances ^30^. A study from Lichand et al. ^31^ suggested the dropout in São Paulo, the richest Brazilian state, may have reached 35% in 2021 in secondary education. As the authors discussed, the consequences of school closures and remote learning during the pandemic might not only affect the already frail learning outcomes but also severely threaten to reverse decades of efforts to ensure universal education in Brazil ^31^. Therefore, we highlight the importance of monitoring and evaluating the long-term consequences of COVID-19 pandemic on school enrollment and learning progress to inform policy and programs actions to mitigate the pandemic-related losses in children and adolescents’ schooling and, consequently, future opportunities ^32^. 

Our study found several individual characteristics related to a greater pandemic impact over life. Adolescents who perceived their health status as regular or poor were 93% more likely to report a greater impact of the pandemic compared to those who perceived their health as excellent, very good, or good. This indicates that perceived health, which may reflect preexisting psychological or physical health condition, is an important factor in determining the extent to which adolescents felt vulnerable by the pandemic. In fact, previous research indicate that individuals with lower self-perceived health status tended to experience greater anxiety and fear about COVID-19 ^33^ ^,^ ^34^. Studies with general population have shown these individuals are more pessimistic about the pandemic’s resolution and more likely to perceive a higher risk of contracting severe forms of COVID-19 ^33^. The fear and anxiety associated with poor health perceptions can amplify the psychological burden of the pandemic, influencing mental health outcomes such as anxiety and depression ^34^. 

Additionally, stricter social distancing measures were associated with a higher likelihood of reporting significant pandemic impact, reflecting the adverse effects of isolation, limited social interactions, and disruptions to daily routines ^35^ ^,^ ^36^. The psychological distress linked to social distancing, as noted by Ammar et al. ^35^, includes increased feelings of isolation and anxiety, which can be especially pronounced in vulnerable populations, such as adolescents. Such findings are consistent with broader impacts observed in various age groups, including older adults, as shown in the study by Greenblatt-Kimron et al. ^36^, emphasizing the widespread effects of social distancing on mental health.

Regarding weight change, while the results were marginally significant, there was some indication that adolescents who gained weight during the pandemic reported they had a more negative perception of its impact. Several risk factors related to weight change during the pandemic, including physical inactivity, poor-quality diet, changes in the eating behavior, and financial difficulties, leading to an increase in the population’s weight gain and in the prevalence of obesity ^37^ ^,^ ^38^. More importantly, a systematic review by Madigan et al. ^39^ highlights the pandemic intensified the adolescents’ exposure to weight stigma content via an increase in social media consumption, affecting their subjective body satisfaction and shaping their psychological well-being. Although we did not ask specific questions about social media usage and/or body image, we found that nearly 80% of girls and 70% of boys reported a negative perception of their use of electronic devices during the pandemic. According to a recent meta-analysis of 46 studies including more than 29,000 children and adolescents, the screen time from before to during the pandemic increased by 52% in average ^39^. With the school closure and limited in-person interactions imposed by the pandemic, it was expected that the adolescents would spend more time using electronic devices as a tool for leisure, online learning, and socializing. However, this surge in screen usage during the pandemic might not be harmful per se. Understanding the adolescents’ subjective judgment of their own screen time can be more informative in identifying the individuals at heightened risk of its harmful effects in their mental health and well-being ^40^. More descriptive and qualitative studies on this matter seem desirable to promote healthy use habits of electronic devices for children and adolescents. Lastly and significantly, 6.4% of the sample, corresponding to nearly 115 adolescents, reported feeling vulnerable for not having access to masks and hygiene products. In the context of a global pandemic, not having access to the best safety measures to protect oneself against contagion highlights the pre-existing socioeconomic disparity in the country, further exacerbated by the pandemic, demonstrating a severe level of insecurity among the population ^40^.

The strengths of this study include a large sample derived from a birth cohort, with information collected via in-person interviews, and the adolescents’ report on several domains of their life. However, we acknowledge that our study also has several limitations. Whilst our sample is large and diverse, it is not representative of the entire Brazilian population. Brazil is a continental country with over 200 million inhabitants and a marked social and geographical inequality, accentuated by the mismanagement of the federal government during the COVID-19 pandemic ^40^. Therefore, the experiences and reports of adolescents from different regions of Brazil may be striking and contrasting. Additionally, the assessment occurred between August and December of 2021, when a considerable fraction of the population was given at least one dose of the vaccine, and the cases and deaths were in a downward trend in Brazil ^2^. If the interviews were conducted in the acute phase of the pandemic, the inputs might have been different. Finally, although our findings have interesting insights into adolescents’ experiences during the pandemic, we could not deeply investigate the topics. We encourage future qualitative studies to comprehensively explore the adolescents’ perception over the COVID-19 pandemic and provide profound and detailed information. 

This study findings highlight the substantial impact of the COVID-19 pandemic on adolescents, with a significant proportion reporting disruptions in multiple aspects of their lives. Notably, the effects were more pronounced among female adolescents, private school students, those who did not receive government financial assistance, and individuals with poorer self-reported health status. The results emphasize the role of socioeconomic, family, and individual factors in shaping adolescents’ experiences during crises. The heightened vulnerability of specific groups underscores the need for targeted interventions and policies to mitigate long-term consequences. Understanding adolescents’ perspectives is essential for informing post-pandemic recovery strategies and fostering resilience in future public health emergencies.
